# Physiological Functions and Pathological Roles of PXR

**DOI:** 10.1210/jendso/bvaf119

**Published:** 2025-07-12

**Authors:** Kazuhiro Ikeda, Kuniko Horie, Satoshi Inoue, Bruce Blumberg

**Affiliations:** Division of Systems Medicine and Gene Therapy, Saitama Medical University, Hidaka-shi, Saitama 350-1241, Japan; Division of Systems Medicine and Gene Therapy, Saitama Medical University, Hidaka-shi, Saitama 350-1241, Japan; Division of Systems Medicine and Gene Therapy, Saitama Medical University, Hidaka-shi, Saitama 350-1241, Japan; Department of Systems Aging Science and Medicine, Tokyo Metropolitan Institute for Geriatrics and Gerontology, Itabashi-ku, Tokyo 173-0015, Japan; Department of Developmental and Cell Biology and Biomedical Engineering, University of California, Irvine, CA 92697-2300, USA

**Keywords:** PXR, detoxification, metabolic homeostasis, inflammation, immune responses

## Abstract

The pregnane X receptor (PXR, NR1I2), a member of the nuclear receptor superfamily, mainly acts as a ligand-activated transcription factor. PXR is predominantly expressed in the liver and intestines, though it is also present at lower levels in various other tissues. PXR is known for its critical role in regulating the metabolism of various chemical substances, including dietary, xenobiotic, and endogenous compounds. As a master regulator of detoxification pathways, PXR modulates the expression of drug-metabolizing enzymes and transporters, contributing to the clearance of potentially harmful compounds. Beyond this classic role, emerging evidence highlights a broader role for PXR on metabolic homeostasis and its involvement in several physiological processes and diseases. This review provides a comprehensive overview of PXR functions across several key metabolic pathways. PXR influences glucose homeostasis by modulating the expression of genes involved in glucose production and insulin sensitivity, highlighting its important role in glucose regulation. PXR regulates the synthesis, breakdown, and transport of lipids, and regulates the expression of genes involved in fatty acid oxidation and cholesterol homeostasis. PXR is involved in inflammatory diseases by modulating the expression of inflammatory cytokines and immune responses, indicating that PXR is a potential target for therapeutic interventions in inflammatory disorders. Furthermore, we discuss PXR function on the regulation of vitamin, bone, and bile acid metabolism.

Pregnane X receptor (PXR; NR1I2), also known as steroid and xenobiotic receptor (SXR), is a ligand-modulated transcription factor belongs to the nuclear receptor (NR) superfamily, first identified in 1998 [1] and named for its ability to be activated by endogenous 21-carbon pregnane steroids [2]. PXR is primarily expressed in organs such as the small intestine, liver, rectum, colon, and bladder, while its presence in other tissues and organs is either moderate, low, or undetectable [3]. PXR can be activated by various chemical compounds, including pregnanes, steroid hormones, bile acids, and other endobiotics, clinically useful drugs (eg, statins, antidepressants, anticonvulsants), and environmental pollutants [4]. The broad ligand spectrum of this receptor is due to its much larger and more adaptable ligand-binding pocket compared to other NRs [5].

The PXR protein consists of several domains: the N-terminal ligand-independent activation function 1, the DNA binding domain, a short hinge region, and the ligand binding domain (LBD) that contains the ligand-dependent activation function 2 domain [6]. Although some reports suggest that PXR is primarily retained in the nucleus [7], PXR can translocate from the cytoplasm to the nucleus on ligand binding, forming a heterodimer with retinoid X receptor (RXR) to initiate transcription of target genes. The unique large and flexible structure of the LBD allows PXR to bind various hydrophobic ligands, making it a versatile receptor [8]. Importantly, there are considerable species differences in PXR ligand specificity between humans and mice. While numerous ligands for human PXR (hPXR) have been identified, including rifampicin, rifaximin, and statins, the range of known ligands for mouse PXR (mPXR) remains limited. Notably, rifampicin, a well-known hPXR ligand, does not activate mPXR, and pregnenolone 16α-carbonitrile (PCN), a typical mPXR activator, has little effect on hPXR. In addition, it has been suggested that the ligand of either PXR or RXR can influence the activity of the partner receptor. For example, the RXR ligand 9-*cis* retinoic acid has been reported to enhance the transcriptional activity of the PXR/RXR heterodimer [9]. However, further research is needed to elucidate the detailed mechanisms specific to the PXR/RXR heterodimer.

Posttranslational modifications are critical in modulating PXR activity. Phosphorylation, acetylation, SUMOylation, poly (ADP-ribosyl)ation, and ubiquitination can cause dynamic changes in PXR's biological properties, including its subcellular localization, dimerization, stability, interaction with coregulators, and degradation [10]. Protein kinases such as protein kinase A have been shown to phosphorylate hPXR, mostly reducing its activity by retaining it in the cytoplasm and preventing it from binding to nuclear DNA, thus suppressing the transcription of downstream genes [11, 12].

In addition to its role in regulating drug metabolism and disposition by modulating the expression of drug-metabolizing enzymes and transporters [8], PXR was recently shown to regulate various physiological and pathological processes, contributing to disease development [13]. In this review, we focus the range of PXR's functions, with an emphasis on its roles in metabolism and related diseases (Fig. 1).

**Figure 1.**
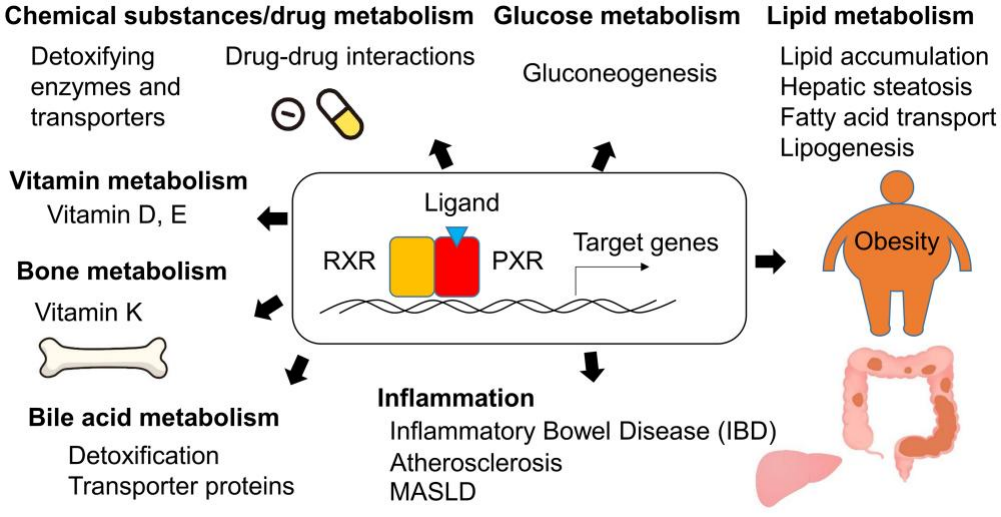
Physiological functions and pathological roles of pregnane X receptor (PXR). In addition to its key role in regulating xenobiotic metabolism, PXR has crucial roles in managing glucose and lipid metabolism, controlling inflammation, metabolizing vitamins and steroid hormones, and detoxifying bile acids.

## Pregnane X Receptor Regulates Metabolism of Chemical Substances and Drugs

PXR directly regulates the expression of drug-metabolizing enzymes, such as CYP3A4, CYP2C9, CYP3A7, and UDP glucuronosyltransferase family 1 member A1 (UGT1A1), along with drug transporters like adenosine triphosphate (ATP) binding cassette subfamily C member 2 (ABCC2), and multidrug resistance 1 (MDR1, aka ABCB1) [14]. The CYP3A family is vital for drug biotransformation, with CYP3A4 being responsible for metabolizing over half of the drugs currently in clinical use [15]. Drugs commonly prescribed, including dexamethasone (DEX), clotrimazole, and rifampicin, induce CYP3A4 expression by activating PXR directly as agonist (clotrimazole and rifampicin) and indirectly through other receptors such as glucocorticoid receptor (GR) (DEX), leading to drug-drug interactions in which one drug accelerates the metabolism of another, potentially causing adverse effects [16]. A well-documented example is the interaction between rifampicin and the immunosuppressant cyclosporine, primarily metabolized by CYP3A4 in the liver [17].

## Pregnane X Receptor Regulates Glucose Metabolism

Accumulating evidence supports the role of PXR in glucose homeostasis, in which it downregulates gluconeogenic genes, including glucose-6-phosphatase (G6Pase) and phosphoenolpyruvate carboxykinase (PEPCK) [18, 19]. hPXR is associated with the cyclic adenosine monophosphate (cAMP) response element-binding protein (CREB), reducing cAMP-mediated transcription of G6Pase [18]. hPXR interacts with the peroxisome proliferator-activated receptor γ (PPARγ) coactivator 1α (PGC-1α), downregulating PEPCK by disrupting PGC-1α interaction with hepatocyte nuclear factor 4, thereby affecting glucagon action [20]. PEPCK and G6Pase downregulation also occurs in response to PCN administration in high-fat diet (HFD)-fed mice [21]. PXR can reduce blood glucose levels through the suppression of gluconeogenesis, making it a promising new therapeutic target for the prevention and treatment of obesity and type 2 diabetes.

PXR also influences oxidative metabolism of glucose. In rats and mice, PCN treatment downregulated glucose transporter 2 (*GLUT2*) and glucokinase (*GCK*) messenger RNAs (mRNAs) [22, 23], suggesting that mPXR impairs glucose tolerance. Dysregulation of GLUT2 and GCK may contribute to mPXR-induced postprandial hyperglycemia, as shown by hepatic deficiency of GLUT2 and GCK in mice with normal feeding [24, 25]. hPXR can mediate statin-induced inhibition of glucose uptake and consumption in HepG2 cells by downregulating GLUT2 and GCK [26]. In this context, it is notable that antidiabetic drugs such as thiazolidinediones, which are PPARγ agonists used to treat insulin resistance, can activate PXR, potentially causing adverse metabolic effects or insulin resistance via interfering with PPARγ signaling [27].

Interestingly, *Pxr* knockout (KO) mice fed an HFD did not become obese or insulin-resistant compared with controls; this was attributed to increased oxygen consumption and energy expenditure [28]. Therefore, PXR appears to function in a manner that represses glucose metabolism. Adipocyte-specific loss of *Pxr* did not affect HFD-induced obesity and metabolic phenotypes, suggesting that PXR signaling in adipocytes is not important for diet-induced obesity and metabolic disorders in mice [29]. Additionally, a recent report indicated that PCN did not affect insulin sensitivity or glucose uptake in skeletal muscle, in addition to exhibiting minimal transcriptomic changes [30]. These results may be attributable to low expression of PXR in the tissue. Overall, PXR plays a key role in hepatic glucose regulation in addition to its known functions in xenobiotic metabolism, suggesting its clinical potential in managing diabetogenic mechanisms and glucose metabolism.

## Pregnane X Receptor Regulates Lipid Metabolism

### Hepatic Lipid Accumulation and Steatosis

Previous studies emphasized the relevance of PXR in regulation of lipid metabolism [4, 31, 32]. Transgenic mice with constitutively active hPXR exhibited a pronounced increase in liver steatosis, primarily due to hepatic triglyceride accumulation [33]. The human-selective PXR agonist rifampicin also induced steatosis in PXR-humanized mice [34]. Moreover, mPXR activation by PCN led to systemic hypertriglyceridemia in mice [35]. Hepatic lipid accumulation dependent on activated PXR is likely influenced by multiple biological processes. For instance, transgenic mice expressing an activated form of hPXR exhibited intrahepatic triglyceride accumulation, together with the upregulation of CD36, a free fatty acid (FA) transporter that facilitates FA uptake from the bloodstream [33], potentially contributing to hepatic steatosis [36]. hPXR directly regulates CD36 transcription [33] and indirectly does so by inducing expression of PPARγ [37].

### Lipogenesis and Lipoprotein Balance

PXR induces key factors in lipogenesis such as fat synthesis-related thyroid hormone–responsive spot 14 protein (S14) [38], lipin-1, which increases triglyceride synthesis [28], and solute carrier family 13 member 5 (SLC13A5), which modulates FA and cholesterol synthesis [39]. PXR also regulates β-oxidation and ketogenesis, particularly by downregulating enzymes such as carnitine palmitoyltransferase 1 (CPT1a) and 3-hydroxy-3-methylglutaryl-CoA synthase (HMGCS2) [35].

PXR is also crucial for lipoprotein balance maintenance, dysregulation of which may cause dyslipidemia. PXR activation in mice substantially elevated overall cholesterol levels and atherogenic lipoproteins low-density lipoprotein (LDL) [40]. In addition, PCN was demonstrated to decrease high-density lipoprotein (HDL) in a transgenic mouse model possessing human-like lipoprotein metabolism [41]. PXR can affect HDL metabolism by downregulating genes responsible for HDL production, maturation, and clearance [41, 42].

#### Obesity-related metabolism and estrogen

Comparison between wild-type (WT) and *Pxr*-KO mice fed an HFD or normal chow diet for 16 weeks indicated that PXR deficiency reduced the HFD-induced body weight gain both in females and males. However, parameters related to metabolic syndrome symptoms were not significantly affected in HFD-fed female WT mice, in contrast to their male counterparts. These findings are consistent with a sex-specific role for PXR in metabolic regulation, contributing to obesity-related disorders exclusively in males but not in females in short-term HFD [31]. A study in which mice were fed an HFD for 52 weeks showed that body and liver weights, as well as hepatotoxicity markers, were significantly elevated in WT mice compared to their *Pxr-*KO counterparts, and PXR deficiency in females protected against severe obesity and harmful effects [32]. The liver damage observed in HFD-fed WT female mice was associated with increased hepatic expression of mRNAs encoding *Pparg* and its downstream genes, such as fat-specific protein 27 (*Fsp27*) and the liver-specific isoform *Fsp27b*. Conversely, *Pxr-*KO mice exhibited higher hepatic levels of antiobesity and anti-inflammatory genes like *Cyp2a5*, *Akr1b7*, glutathione-S-transferase M3 (*Gstm3*), and AMP-activated protein kinase (*Ampk*). RNA-sequencing revealed that HFD activated pathways including oxidative stress, cholesterol biosynthesis, and glycolysis/gluconeogenesis in WT mice, whereas these pathways were not activated in *Pxr*-KO mice. Thus, PXR promotes HFD-induced obesity in mice, with a relatively modest effect in females. The long-term HFD study may raise another question whether estrogen modulates PXR functions. A study in ovariectomized female mice showed that HFD-induced weight gain and adipose tissue inflammation were prevented by estrogen supplementation [43]. An inverse relationship between PXR and estrogen receptor α (ERα) was observed in endometrial cancer [44], which is predominantly attributable to obesity. ERα-KO in liver cells makes females susceptible to triglyceride accumulation and insulin resistance [45]. While HFD significantly reduced hepatic ERα mRNA levels in WT mice, this was not observed in *Pxr*-KO mice. Therefore, it is plausible that PXR suppresses estrogen/ERα signaling, exacerbating obesity and liver toxicity in HFD-fed WT mice. In contrast, PXR deficiency preserves ERα expression, offering protection against fatty liver disease.

#### Pregnane X receptor ligands and lipid metabolism

Lipid metabolism is also affected by PXR ligands such as the antiepileptic drug valproate (VPA). VPA is widely used in the treatment of epilepsy; however, long-term use has been associated with adverse effects such as hepatic steatosis, fatty liver disease, and obesity [46]. It has been demonstrated that VPA promotes lipid accumulation in HepG2 hepatocellular carcinoma cells [47]. Mechanistically, gain- and loss-of-function studies revealed that VPA increases the expression of PXR and FA binding protein 4, a key molecule involved in FA transport, which is required for VPA-induced lipid accumulation [47]. Perinatal exposure to the endocrine-disrupting chemical bisphenol A (BPA) exacerbated atherosclerosis in adult male PXR-humanized and ApoE KO (hPXR•ApoE^−/−^) mice, because BPA-induced PXR activation epigenetically regulates FA transporter CD36 expression [48]. Antiretroviral drug efavirenz-induced PXR activation regulates cholesterol biosynthetic enzyme squalene epoxidase (SQLE) and CD36, resulting in increased lipid uptake and cholesterol synthesis in mouse hepatocytes. This suggests that long-term use of PXR-activating antiretroviral drugs may cause hypercholesterolemia and steatosis [49]. Another antiretroviral drug, cobicistat, exhibits PXR-mediated hepatotoxicity through CYP3A4-dependent pathways [50].

#### Pregnane X receptor phosphorylation and lipid metabolism

PXR phosphorylation is essential in lipid metabolism as it prevents fasting-associated hepatotoxicity, clarified by a recent study of mice carrying the PXR∗Ser347Ala knockin mutation (PXR-KI) [51]. Serine (Ser) 347 residue in the LBD of mPXR (corresponds to Ser350 in hPXR) was identified as a phosphorylation site responsive to fasting conditions [52]. Compared to PXR-WT mice, the PXR-KI mice exhibited a phenotype with reduced body weight overall and increased body weight loss during fasting. In terms of lipid metabolism, hepatic triglyceride accumulation was prominent in the fasting PXR-KI mice. The effect of fasting on adipose tissue lipolysis is critically important, as it promotes the breakdown of stored triglycerides into free FAs and glycerol. Although the PXR-KI mouse is a systemically gene-modified model, its effect on adipocyte function has not been fully elucidated. Further studies are needed to clarify the precise role of PXR phosphorylation in hepatic triglyceride accumulation during fasting.

Phosphorylation at Ser350 in hPXR disrupts its heterodimerization with retinoid X receptor (RXR)α [53]. Considering that PXR also interacts with other transcription factors involved in glucose and lipid metabolism—such as FOXO1, FOXA2, PPARα, CREB, and PGC-1α [18, 35, 54, 55]—it is possible that phosphorylation of PXR at Ser347 in mice (Ser350 in humans) may affect the functions of these protein partners, thereby influencing target gene expression in response to energy stress. Although this remains to be clarified, it could be an interesting subject for future studies.

It is notable that PXR ablation was associated with the development of hepatic steatosis and accumulation of lipids and triglycerides during fasting [35], consistent with the loss of phosphorylated PXR being involved in the liver disorders. Fasted PXR-KI mice, unable to be phosphorylated at Ser347, showed elevated expression of genes in metabolic pathways related to estrogen biosynthesis, nicotine degradation, melatonin degradation, and glutathione-mediated detoxification [51]. Activation of these pathways was also observed in mice treated with PXR ligands [56]. Among the metabolic pathways, nicotine degradation and glutathione-mediated detoxification are linked to liver detoxification system whereas estrogen exerts a protective effect against liver lipid accumulation [45]. These findings indicate that PXR and its phosphorylation at Ser347 play a role in protecting the liver both from toxic and energy-related stresses.

PXR undergoes various posttranslational modifications, including phosphorylation, SUMOylation, ubiquitination, and acetylation at different amino acid residues [12, 57]. These modifications can influence its transcriptional activity and function as a mechanism for crosstalk between different signaling pathways, suggesting a potential role in lipid metabolism. However, the physiological relevance of these modifications remains to be fully elucidated.

## Pregnane X Receptor Regulates Inflammation and Inflammatory Diseases

### Inflammatory bowel disease

PXR deficiency or reduced expression has been observed both in inflammatory bowel disease (IBD) patients and mouse models, suggesting that impaired PXR signaling may contribute to the pathogenesis of intestinal inflammation. In humans, PXR expression is found to be downregulated in the gastrointestinal tract of patients diagnosed with ulcerative colitis [58]. Furthermore, the administration of rifampicin has been observed to induce PXR expression, concomitant with a decline in nuclear factor-κB target genes, including interleukin-8, in biopsy samples from active Crohn disease patients [59]. In murine models, PXR deficiency has been demonstrated to contribute to nuclear factor-κB–driven small intestinal inflammation [60]. These findings imply that PXR status plays a pivotal role in the regulation of inflammation in IBD.

PXR plays a key role in regulating intestinal homeostasis [61]. Human MDR1 (ABCB1), a member of the ABC transporter family, is primarily controlled by PXR and highly expressed in intestinal epithelial cells [62]. The *MDR1* gene encodes the transmembrane protein P-glycoprotein (P-gp), which uses ATP to expel toxins from enterocytes back into the gut lumen, thereby preventing the accumulation of drugs and other substrates in cells and helping to maintain intestinal homeostasis [62]. PXR downregulation is likely the main cause of the subsequent reduction in *MDR1* expression during intestinal inflammation [58]. Additionally, IL-1β, which has a role in inflammation and immune response in IBD, reduces both expression and functionality of P-gp in human intestinal epithelial cells [63]. Notably, imidacloprid treatment downregulates PXR, leading to elevated tumor necrosis factor-α (TNF-α) and IL-1β levels in vitro and in vivo [64]. Furthermore, PCN prevents dextran sulfate sodium–induced colitis by inhibiting TNF-α and IL-1β via a PXR-dependent pathway [65]. This suggests an inverse relationship between PXR and IL-1β in the regulation of intestinal homeostasis, particularly in the context of IBD. In summary, the reduction of PXR expression and activity in inflamed intestinal epithelial cells leads to downregulation of MDR1/P-gp, contributing to disrupted intestinal homeostasis.

PXR deficiency plays a key role in driving the disruption of intestinal epithelial barriers and increasing intestinal permeability in IBD. *Pxr-*KO mice showed pronounced ultrastructural defects including: a reduction in the villus-crypt ratio, shorter microvilli, and increased electron density in the tight-junction and adherens-junction complexes within the intestinal epithelium [66]. These mice also exhibited heightened intestinal permeability, thus the absence of PXR worsens the disruption of intestinal barrier integrity. The mechanism through which PXR influences intestinal barrier function and permeability likely involves toll-like receptor 4 signaling [67].

In a recent study of the intestinal epithelial-specific *Pxr*-KO (i*Pxr*-KO) mice, i*Pxr*-KO mice displayed normal intestinal permeability compared to control mice and histological analysis of the jejunum showed no differences between the two groups [68]. Even after treatment with lipopolysaccharide, the overall health status of i*Pxr*-KO and the control mice remained comparable. The results suggest that targeted deletion of *Pxr* in the intestinal epithelium does not lead to significant intestinal damage in mice, either under steady-state or inflammatory conditions. In contrast to i*Pxr*-KO mice, fibroblast-specific *Pxr*-KO mice mimicked the intestinal phenotype of *Pxr*-KO mice with increased inflammation and fibrosis, thus demonstrating the importance of PXR expression in nonepithelial cells for the maintenance of intestinal homeostasis after injury [68]. While the role of PXR in IBD pathogenesis is complex, further research is necessary to better understand its novel functions and underlying molecular mechanisms.

#### Atherosclerosis

Atherosclerosis is a chronic inflammatory condition characterized by the excessive accumulation of LDL cholesterol in the arterial walls. The role of PXR in atherosclerosis remains controversial since studies have reported both proatherogenic and antiatherogenic effects. These discrepancies may suggest that PXR function in atherosclerosis is dependent on different factors such as species, PXR ligands, and experimental conditions as summarized next.

Reduction of atherosclerosis was shown in LDL receptor–deficient mice with myeloid-specific PXR deficiency [69]. Perinatal exposure to BPA led to atherosclerosis in adult male PXR-humanized mice [48] and PXR-humanized ApoE-deficient mice [70]. Supporting these findings, PXR activation induced hypercholesterolemia in WT mice and exacerbated atherosclerosis in ApoE-deficient mice [40], whereas PXR deficiency reduced atherosclerosis in ApoE-deficient mice [71]. The harmful effects of PXR were linked to reduced antiatherogenic ApoA-IV levels in the liver [71].

PXR deletion reduced foam cell formation and lipid accumulation in macrophages [69], which are central processes for atherosclerosis development [72]. PXR ablation also downregulated CD36, resulting in decreased uptake of oxidized LDL [48, 69]. PXR modulates the expression of several genes such as the cholesterol absorber Niemann-Pick C1-Like 1 (*Npc1l1*) in macrophages [69].

On the other hand, PXR ligands can inhibit platelet functions (aggregation, adhesion, and granule secretion) and reduce thrombus formation through nongenomic mechanisms [73]. The human-specific PXR ligand SR12813, an agonist with respect to genomic function, decreases thrombus formation in vivo in humanized PXR transgenic mice by inhibiting Src-family kinases [73]. Thus, the antithrombotic effects of PXR ligands may offer additional antiatherosclerotic benefits, although conflicting results were also shown by different studies [40]. Further studies are required to elucidate the precise mechanisms underlying PXR-related atherosclerosis.

#### Metabolism associated fatty liver disease

MASLD is a nonalcoholic steatotic liver disease [74], and its pathogenesis may be closely related with PXR signaling as exemplified by PXR target *Cyp3a11* being upregulated during MASLD progression in mice [75]. PXR-induced hepatic lipogenesis and reduction of lipid oxidation were observed both in mouse models and in human primary hepatocytes [76, 77]. Mechanistically, PCN downregulated the expression of CPT1A (involved in β-oxidation) and mitochondrial HMGCS2 (ketogenesis) while upregulating SCD1 (lipogenesis) in a PXR-dependent manner [35]. Moreover, both genetic and pharmacological activation of PXR in the liver induced the transcription of *Cd36* and its positive regulator *Pparg* [33]. SLC13A5, a transporter of circulatory citrate into hepatocytes, was also induced by PXR and promoted de novo lipogenesis [77]. Interestingly, PXR ablation in mice prevented HFD- and gene mutation–induced insulin resistance and hepatic steatosis [28] while severe spontaneous hepatic steatosis was observed in *Pxr*-KO mice [35], indicating that both the activation and depletion of PXR can induce the development of steatosis. Several NRs are known to exert regulatory functions even in the absence of ligand binding. It has been proposed that unliganded (apo) PXR can repress transcription by recruiting corepressors [78]. Accordingly, apo-PXR may contribute to the repression of genes involved in insulin production and hepatic steatosis. Additionally, another study reported that RXR negatively regulates glucose-stimulated insulin secretion, suggesting that RXR may also play a role in this regulatory axis [79]. However, further studies are needed to clarify the precise mechanisms involved. PXR function in lipid metabolism and MASLD may be also affected by differences in genetic backgrounds and agonist administration conditions. Indeed, some *PXR* polymorphisms were clinically associated with greater disease severity in MASLD [80], or linked to increased mortality risk only in individuals with MASLD [81].

#### Alcoholic liver disease

Sexual dimorphism in PXR function may play a role in the development of alcohol-induced hepatotoxicity. PXR potentiated alcohol-induced liver damage in male mice, whereas female *Pxr*-null mice showed resistance to chronic alcohol-induced hepatotoxicity [82]. Female *Pxr*-KO mice metabolized alcohol faster and showed increased levels of protective liver enzymes and lipid metabolism–related proteins, which help protect against alcohol-induced oxidative stress and liver damage. Interestingly, liver injury from alcohol was similar between male and female WT mice, but WT female mice seemed to be protected from developing macrovesicular steatosis in the liver. In transcriptomic analysis, pathways related to retinol and steroid hormone production, cancer-related chemical metabolism, and arachidonic acid metabolism were upregulated by alcohol in a PXR-dependent way in both sexes. These findings indicated that female *Pxr*-KO mice showed resistance to chronic alcohol-induced liver damage, revealing both PXR-dependent and -independent mechanisms involved in alcohol-related liver toxicity.

## Pregnane X Receptor Regulates Vitamin Metabolism and Bone Metabolism

Vitamin K is crucial for bone formation and has been used in the clinical treatment of osteoporosis in Asian countries [83]. Vitamin K2 activates PXR, promoting the expression of its target genes. In osteoblastic cells, vitamin K2 treatment led to an increase in the expression of osteoblast markers, including bone alkaline phosphatase, osteoprotegerin, bone bridging protein, and scaffold Gla protein [84]. Analysis of bone tissue from PXR-deficient mice revealed that the bone-protective effects of vitamin K2 are partially mediated by PXR-dependent signaling [85]. Vitamin K2 induced the expression of bone markers, MGP and OPG, in primary osteoblasts from WT mice but not in *Pxr* KO mice. Ichikawa et al [86] identified certain PXR target genes that contribute to osteoblast function, including *TSUKUSHI*, *MATN2*, and *CD14*.

Recent studies have reported that PXR inhibits osteoblast differentiation [87, 88]. The researchers investigated global gene expression changes in calvarial osteoblasts cultured under two conditions: standard fetal bovine serum, in which PXR inhibits differentiation, and charcoal-stripped fetal bovine serum, in which PXR does not inhibit differentiation. The authors proposed that PXR diminished Hedgehog-mediated signaling under the culture conditions, leading to PXR-mediated repression of differentiation, suggesting its autonomous effects on osteoblasts. Additionally, PXR is involved in bone mineral homeostasis in vivo: Its activation upregulates the *CYP24* gene, which hydroxylates and inactivates the active form of vitamin D [76, 77].

Vitamin D plays a crucial role in regulating calcium absorption and excretion, with its active metabolite, 1,25(OH)₂D₃, binding to the vitamin D receptor (VDR). On activation, VDR stimulates 24-hydroxylation through the enzyme 25-hydroxyvitamin D₃-24-hydroxylase (CYP24), which is essential for the metabolism of 1,25(OH)₂D₃. Pascussi et al [89] reported that PXR activation upregulated *CYP24* expression, which may explain drug-induced osteomalacia. Conversely, Zhou et al [76] found that PXR activation induced CYP3A4 expression but not CYP24. The authors advocate that CYP3A4 plays a role in inactivation of 1,25(OH)_2_D_3_ in human liver and intestine. These findings highlight the possibility that PXR has complex roles in vitamin D catabolism and drug-induced osteomalacia, which remains to be further explored. Ligand activation of PXR was shown to inhibit the transcription of vitamin D₃ 25-hydroxylase (CYP2D25), a key enzyme in the biosynthesis of 1,25(OH)₂D₃ [90]. This could also explain why long-term use of anticonvulsant drugs, which activate PXR, is associated with osteomalacia [91].

Vitamin E is commonly consumed as an antioxidant in the daily diet. It undergoes metabolism via CYP-mediated oxidative reactions and is excreted through β-oxidation and conjugation reactions, including glucosylation and sulfation [92]. These metabolic pathways involve enzymes and transport proteins regulated by PXR target genes. Tocotrienol forms of vitamin E activate PXR, potentially influencing the expression of genes related to its own metabolism [93]. A study by Landes et al [94], using reporter gene analysis, demonstrated that certain forms of vitamin E can activate PXR. In an experiment with WT mice, levels of vitamin E metabolites in the urine were significantly reduced following PCN treatment, whereas this effect was absent in *Pxr* KO mice. The results suggest that PXR-mediated reduction in hepatic sterol carrier protein 2 expression may lead to decreased β-oxidation [95]. These findings have sparked substantial interest in exploring potential drug-drug interactions between vitamin E and PXR modulators.

## Pregnane X Receptor Regulates Bile Acid Metabolism

Bile acids are synthesized in the liver as the final product of cholesterol breakdown and play a key role in the elimination of cholesterol from the body [96]. Once excreted into the intestine, bile acids aid in the absorption of cholesterol and fat-soluble vitamins. However, an excess of bile acids can be cytotoxic and may result in pathological cholestasis. To prevent toxic damage, bile acid levels must be tightly controlled. PXR is critical in detoxifying bile acids. In WT mice, PCN reduced lithocholic acid (LCA)-induced toxicity, a protective effect not observed in *Pxr* KO mice. Additionally, transgenic mice overexpressing PXR exhibited resistance to LCA toxicity. This protective role of PXR was attributed to its regulation of genes involved in bile acid metabolism. For example, the phase 2 metabolic enzyme SULT2A, a PXR target gene, participates in bile acid detoxification [97]. Beyond regulating bile acid synthesis and metabolism, PXR also influences the expression of bile acid transport proteins such as MRP2 and OATP2 [98, 99]. LCA itself is known to regulate the expression of genes involved in bile acid metabolism in WT mice. For instance, a relatively low-dose, short-term administration of LCA has been reported to increase the hepatic expression of proteins involved in bile acid detoxification and transport, such as Sult2a and Oatp2 [98, 100]. These regulatory effects are suggested to be mediated not only by PXR but also by other NRs, including RXR [101], FXR [102], and VDR [103].

Bilirubin is a breakdown product of hemoglobin. UDP-glucuronosyltransferase (UGT) binds to bilirubin and converts the neurotoxic unconjugated form into nontoxic bilirubin-glucuronide. Activation of PXR in mice was shown to reduce hyperbilirubinemia. Compounds such as oleanolic acid and ursolic acid enhance the transcription of UGT1A1 and other key genes involved in bilirubin detoxification, such as OATP2 and MRP2, via PXR activation [98, 104]. OATP2 helps transport bilirubin from the bloodstream into the liver, while MRP2 aids in excreting conjugated bilirubin into bile ducts. Although PXR was initially identified as a sensor for xenobiotics, the discovery that certain bile acids, including LCA, can act as ligands both for human and mouse PXR has established a connection between PXR and bile acid regulation [105].

PXR upregulation has been shown to enhance the metabolism and excretion of bilirubin in the liver of premature infants [106]. In a rat model, treatment with DEX, a ligand for the GR, increased mRNA levels of *Pxr*, *Ugt1a1*, and *Abcc2* (*Mrp2*) in primary fetal hepatocytes. Knockdown of GR or PXR using small interfering RNA reduced the DEX-induced increases in *Ugt1a1* and *Abcc2* mRNA levels, indicating that DEX effects are mediated through both the GR and PXR in these cells. These findings suggest that administering glucocorticoids prenatally can enhance bilirubin metabolism in the liver of premature infants, potentially helping to prevent neonatal jaundice.

NADPH:P450 oxidoreductase (POR) is crucial for the function of microsomal cytochrome P450 (CYP) enzymes, which play a role in the synthesis of bile acids and steroids, as well as the metabolism of xenobiotics. POR also supports redox enzymes involved in FA and cholesterol pathways. Notably, knockdown of POR significantly downregulated PXR [107]. This led to disruptions in bile acid and cholesterol biosynthesis, with altered levels of cholic acid and chenodeoxycholic acid derivatives. These findings suggest that reduced POR expression may indirectly affect PXR-regulated pathways, highlighting the interconnected nature of POR and PXR-mediated metabolic regulation. However, further studies are needed to determine whether PXR downregulation is a direct mediator of these metabolic changes or a downstream consequence of POR deficiency. The roles of PXR in metabolism and its associated inflammatory diseases are summarized in Table 1.

**Table 1.** Potential role and target genes of pregnane X receptor in metabolism- and inflammation-associated diseases

Disease	Role of PXR	Target genes*^a^*	References
Diabetes/hyperglycemia	Glucose metabolism and insulin sensitivity	*G6Pase*, *PEPCK*, *GLUT2*	[21, 26]
Dyslipidemia	Metabolism and transport of lipids	*CYP3A4*, *APOA1*	[108, 109]
IBD	Regulation of inflammation, immunity, and detoxification	*MDR1*, *TNF-α*, *IL-1β*	[65, 110]
Atherosclerosis	Inhibition of inflammation and lipid accumulation	*APOA4*, *NPC1L1*	[40, 111]
MASLD	Regulation of lipid metabolism	*SCD1*, *CD36*, *SLC13A5*	[33, 39]
ALD	Alcohol metabolism and antioxidative stress	*ALDH1*, *ADH1*	[82]
Osteoporosis	Bone and vitamin D metabolism	*MGP*, *OPG*, *CYP24*	[84, 89]
Cholestasis	Prevention of bile acid accumulation	*UGT1A1*, *SULT2A*, *MRP2*	[99, 112]

Abbreviations: ALD, alcoholic liver disease; IBD, inflammatory bowel disease; MASLD, metabolic dysfunction–associated steatotic liver disease; PXR, pregnane X receptor.

^a^Representatives of PXR-target genes are depicted.

## Future Perspectives

Several key challenges and possibilities can be identified for the future direction of research on PXR in metabolism. PXR is known for regulating the expression of drug-metabolizing enzymes and transporters, but the complete picture of its role in metabolic pathways remains unclear, particularly with respect to how PXR affects the metabolism of endogenous substances (eg, lipids, glucose, and bile acids). A deeper understanding of how PXR selectively recognizes xenobiotics (drugs) and endogenous molecules (eg, bile acids, hormones, vitamin K, and lipid metabolites), and how it regulates different pathways in a sex-dependent manner is required. PXR plays an important role in chronic conditions such as metabolism-associated steatotic liver disease, diabetes, obesity, inflammatory diseases, and osteomalacia/osteoporosis. Future studies are needed to clarify how PXR contributes to the progression of these diseases and how targeting PXR could serve as a therapeutic strategy for such chronic conditions. Furthermore, the development of personalized therapeutic strategies can be expected based on the findings regarding genetic polymorphisms in PXR that influence individual responses to drugs and susceptibility to diseases. Future research into the role of PXR in metabolism holds great potential across multiple fields, including drug development, precision medicine, and chronic disease management, and will bring substantial advancements to these clinical challenges.

## Data Availability

No new data were generated or analyzed in this study.

## Abbreviations

ABCC2: ATP binding cassette subfamily C member 2
ALD: alcoholic liver disease
ATP: adenosine triphosphate
BPA: bisphenol A
cAMP: cyclic adenosine monophosphate
CREB: cAMP response element–binding protein
DEX: dexamethasone
ERα: estrogen receptor α
FA: fatty acid
G6Pase: glucose-6-phosphatase
*GCK*: glucokinase
*GLUT2*: glucose transporter 2
GR: glucocorticoid receptor
HDL: high-density lipoprotein
HFD: high-fat diet
hPXR: human PXR
IBD: inflammatory bowel disease
IL-1β: interleukin-1β
KO: knockout
LBD: ligand binding domain
LCA: lithocholic acid
LDL: low-density lipoprotein
MASLD: metabolic dysfunction–associated steatotic liver disease
MDR1: multidrug resistance 1
mPXR: mouse PXR
mRNA: messenger RNA
NR: nuclear receptor
PCN: pregnenolone 16α-carbonitrile
PEPCK: phosphoenolpyruvate carboxykinase
PGC-1α: PPARγ coactivator 1α
POR: NADPH:P450 oxidoreductase
PPARγ: peroxisome proliferator-activated receptor γ
PXR: pregnane X receptor
PXR-KI: PXR∗Ser347Ala knockin mutation
RXR: retinoid X receptor
SXR: steroid and xenobiotic receptor
TNF-α: tumor necrosis factor-α
UGT1A1: UDP glucuronosyltransferase family 1 member A1
VDR: vitamin D receptor
VPA: valproate
WT: wild-type
